# Rheological Characterization of Marine and Bovine Collagen Peptides/Acetic Acid Slurries Incorporating Hydroxyapatite Nanoparticles

**DOI:** 10.3390/polym17233196

**Published:** 2025-11-30

**Authors:** Mario Milazzo, Claudio Ricci, Eugenio Redolfi Riva, Damiano Rossi, Irene Anguillesi, Maurizia Seggiani, Giuseppe Gallone, Serena Danti

**Affiliations:** 1 Department of Civil and Industrial Engineering, University of Pisa, Largo Lucio Lazzarino, 56126 Pisa, Italy; claudio.ricci@unipi.it (C.R.); damiano.rossi@unipi.it (D.R.); irene.anguillesi@unipi.it (I.A.); maurizia.seggiani@unipi.it (M.S.); giuseppe.gallone@unipi.it (G.G.); 2 The BioRobotics Institute, Sant’Anna School of Advanced Studies, Viale Rinaldo Piaggio, 56025 Pontedera, Italy; eugenio.redolfiriva@santannapisa.it

**Keywords:** nanocomposite slurries, viscoelastic characterization, nanocomposite aging, structural stability, polymer–ceramic composites

## Abstract

The development of collagen-based composite materials for bone tissue engineering requires a comprehensive understanding of their rheological and structural behavior to ensure processability and functional stability. This study investigates the viscoelastic and morphological properties of nanocomposite slurries composed of hydroxyapatite (HA) nanoparticles dispersed in acetic acid solutions of bovine or fish-derived collagen peptides. Frequency and strain sweep tests revealed solid-like behavior and shear-thinning characteristics consistent with printable bioinks. Both formulations yield stresses between 0.7 and 1.5 kPa, values comparable to those reported for 3D-printable HA composites. Over ten days of aging, fish-based formulations retained higher viscosity and modulus, indicating improved temporal stability relative to bovine-based ones. Drop-casting tests confirmed the formation of homogeneous, highly opalescent films, with surface profilometry showing lower waviness for the fish-derived blend, suggesting enhanced microstructural uniformity. These results demonstrate that acetic acid-mediated collagen–HA interactions generate stable, high-fidelity slurries suitable for additive manufacturing applications. The superior rheological properties of fish collagen formulations highlight the influence of peptide source on network evolution, offering valuable insight for optimizing collagen–ceramic composites in regenerative and biomedical applications.

## 1. Introduction

Collagen is a natural polymer abundant in human and animal tissues. It is the main constituent of the musculoskeletal system and is the main building block of tendons, ligaments, cartilage and, in particular, bones, in which it is combined with hydroxyapatite (HA) [1,2].

For their bioactive properties, collagens and their derivatives are considered unique biomaterials in the bioengineering field concerning, but not limited to, soft tissue augmentation and wound dressing. Tissue engineering has taken advantage of collagen for repairing damaged or diseased tissues [3,4,5,6,7,8,9]. In particular, collagen has been typically used as the main constituent of scaffolds in combination with cells and/or growth factors (e.g., transforming growth factor beta 1, platelet-derived growth factor) to create provisional biocompatible structures that are able to support and promote the growth of new tissues and to transfer and dissipate energy from external stationary and transient loads [10,11,12,13].

The recent advancements in additive manufacturing techniques, including three-dimensional (3D) printing, have opened new research avenues to explore the employment of collagen in biomedical devices. Composites made of collagen and HA have been widely manufactured using either molding or extrusion-based 3D printing to mimic bone tissues [14]. Therefore, the increasing request for collagen has pushed scientists to search for additional sources other than human tissues. However, ethical and safety issues, strictly related to the extraction and processing of collagenous raw materials, have contributed to increasing the costs of the raw materials or, as an alternative, to the use of recombinant human collagen.

As a matter of fact, commercially available collagen-based biomaterials nowadays are usually of animal origin, mostly mammalian. Bovine collagen (BC) and its derivatives, such as gelatin and collagen peptides, have been commonly used to prepare scaffolds due to their excellent biocompatibility and ability to promote cell proliferation and reorganization by inducing various bioactive signals to express tissue-specific genes [15]. However, BC presents three main issues: (1) lack of reproducibility that is due to physiological conditions and age of the donor; (2) biological reliability since BCs are prone to contamination, e.g., the transmissibility of spongiform encephalopathy agents that are difficult to detect and remove from animal tissues [16]; and (3) recent studies have reported hypersensitivity reactions following implants with bovine collagens [17,18,19,20,21]. Despite these concerns, BC is still widely employed because of its availability and low cost, thus driving scientific research to improve the quality of the tissue.

An alternative source of animal-based collagens is offered by the marine environment. Fish collagen (FC) could be an appropriate alternative to mammalian collagen for regenerative medicine applications, thanks to its biological properties similar to mammalian collagens, lower cost, and minor ethical issues, also providing a relatively low immunogenic response [15].

FC can be isolated from invertebrate and vertebrate marine organisms and can be classified in two main categories based on the original source, usually from food waste: invertebrate collagen (e.g., sponges, jellyfish and shellfish), and fish collagen (e.g., shark, tuna, salmon) [20,21,22,23,24]. From a chemical perspective, there are mainly five types of FC, namely collagen types I (the most abundant), II, IV, V, and XI [23]. The employment of FC has gained attention for TE applications since it has been revealed to be similarly or even more biocompatible than BC [25]. Additionally, it is free from religious limitations [15,22,26]. Moreover, it has been observed that, compared to mammalian sources, FC can be resorbed more efficiently and safely by the body [27].

Zhou et al., for instance, developed a double-layer composite scaffold based on FC, characterized by small pores in the upper layer and larger pores in the lower layer, aiming at mimicking the complex structures of cartilage and bone morphologies. They showed that this scaffold can provide a microenvironment ideal for chondrogenic and osteogenic differentiation of bone marrow stem cells in vitro, which led to simultaneous repair of cartilage and subchondral bones in a rabbit osteochondral defect model [15]. However, despite its great potential, FC revealed several practical problems, such as the challenging control of its biodegradation in vivo, low cell seeding efficiency, and poor mechanical properties [15,25]. To address these issues, composite materials made of FC scaffolds have been designed and proposed in biomedical applications.

Elango et al. used FC to find an alternative to mammalian collagen with adequate osteogenic properties. Two types of composite collagen scaffolds (i.e., collagen/chitosan and collagen/HA) were prepared from blue shark cartilage to compare their physical–functional and mechanical properties, as well as their biocompatibility and osteogenesis features, in relation to those related to mammalian-based collagens, revealing the key role of chitosan in improving viscosity, stiffness, and biodegradation rate of the scaffolds; on the other hand, chitosan worsened scaffold shrinkage and porosity [22]. Controlling all these parameters is of utmost importance to obtain accurate results when using additive manufacturing technologies, as they determine the functional outcome of the produced device.

In a recent study, Milazzo et al. explored the possibility of using sodium alginate/hydroxyapatite-based bioinks incorporating fish- and bovine-derived collagen peptides dissolved in deionized water for bone tissue 3D printing applications. The authors demonstrated comparable chemical structures of the peptides and marked solid-like rheological behavior suitable for extrusion printing. In detail, fish-based inks exhibited higher viscosity and better geometry retention, while bovine-based inks showed improved pore definition and swelling capacity. Both formulations enabled fabrication of accurate and stable scaffolds and supported human fibroblast viability, highlighting their potential as eco-friendly materials for bone tissue engineering [28].

Differently from previous study, our work addresses the possibility of using nanocomposite slurries made of animal collagen peptides, dissolved in acetic acid, and reinforced with HA particles. We present a systematic rheology study on the aging of such materials also in relation to their employment in additive manufacturing applications. Finally, we show the possibility of using the nanocomposite slurries using drop casting, one of the multiple options in additive manufacturing [14], including a quantitative analysis of the obtained morphology for the production of biocompatible biomedical devices.

## 2. Materials and Methods

### 2.1. Materials

Collagen peptides from fish and bovine sources (average M_w_ = 2 kDa), in the form of powder, were donated by Lapi Gelatine S.p.A. (Empoli, FI, Italy). HA in the form of powder (average diameter < 1 µm, M_w_ = 502.31) was purchased from Acros Organics (Fisher Scientific, Hampton, NH, USA). Glacial acetic acid (AA) (concentration equal to 96% *v*/*v*) was purchased from Sigma-Aldrich (Merck KGaA, Darmstadt, Germany).

### 2.2. Preparation of the Inks

A total of 30 g of HA and 17.5 g of collagen peptides were weighed and collected in a glass beaker. While gently stirring (ω = 50 rpm) the powder with a mechanical agitator made of Teflon, we progressively added acetic acid to achieve a HA/collagen peptides/AA ratio of 1.7:1:1 (*w*/*w*/*v*), a composition that led to a full absorption of the powder and the formation of a viscous ocher substance. The same preparation was performed for both the BC/HA and FC/HA samples at room temperature.

### 2.3. Rheology Tests

Rheology tests were performed using the MCR 92 Rheometer (Anton Paar GmbH, Graz, Austria) equipped with a 25-mm parallel plate tool. To avoid friction between the blend and the experimental equipment, we used a 50-µm fixed gap between the two steel plates. While reaching the correct gap distance, the excess of material was carefully removed using a dedicated tool.

Preliminary tests on the samples were performed to evaluate the linear–elastic interval for the investigated compositions. Thereafter, effective measurements were carried out to estimate the main rheological properties, namely the amplitude of the complex viscosity (*η*), the storage (*G′*) and loss (*G″*) shear moduli, and the evolution of the shear stress (*τ*) as a function of either the shear strain (*γ*), or the input frequencies (*f*), or the shear rate ($\dot{\gamma }$). The number of samples was fixed at *n* = 6 (repeated measurements taken for each formula/time point); therefore, plots in the Results section will show the average value of the measurements. The measured quantities are linked together by the following constitutive equations:

(1) τ=G·γ

(2) $$\tau =\mu \cdot \dot{\gamma }$$

with both the Shear Modulus, *G*, and the viscosity, μ, being complex quantities, i.e.:

(3) $$G=G^{\prime }+j\cdot G^{\prime \prime }$$

(4) $$\mu^{*}=\mu^{\prime }-j^{\prime \prime }$$

where $j^{2}=-1$. This implies that, for a harmonic stimulus *γ(t)* at any given frequency *f*,

(5) $$\mu^{*}=G/\omega$$

where $\omega \left[\mathrm{rad}/s\right]\;=2\pi f$ is the corresponding angular frequency. It is important to note that the quantity $\eta^{*}$, which is reported in the graphs of this work and discussed throughout this paper, actually represents the mathematical modulus of the complex viscosity

(6) $$\eta =\left|\mu \right|=\sqrt{{\mu^{\prime }}^{2}+{\mu^{\prime \prime }}^{2}}\;\;$$

The relationship between the frequency and the shear rate is defined as follows:

(7) $$\dot{\gamma }=2\pi f\frac{R}{H}$$

where *R* is the radius of the parallel plates used in the rheological characterization, which in our case was equal to 12.5 mm, and *H* is the gap set between the plates, in our case equal to 50 µm [29]. In order to characterize the stability of the material with time, we performed sweep tests (both a frequency sweep and a strain amplitude sweep for each of the two studied formulations) with three checkpoints in a 10-day framework, namely day 1, day 6, and day 10. Frequency sweep tests were carried out by considering a fixed strain amplitude of 0.05% and a frequency bandwidth between 0.1 Hz and 100 Hz (shear rate between 157 s^−1^ and 157,000 s^−1^). Strain sweep tests were performed using a fixed frequency of 1 Hz and strain amplitudes ranging from 2 × 10^−3^ to 2%.

We defined specific rheological parameters from the amplitude sweep study as follows: *η*_0_ and *G′*_0_, i.e., viscosity and storage modulus at the lowest measured strain, and *τ_y_* and *γ_y_*_,_ i.e., yield stress and yield strain.

### 2.4. Morphological Analysis of Drop-Casted Samples

The nanocomposite slurries were stored at 4 °C after preparation to avoid solvent evaporation. A custom-made polytetrafluoroethylene (PTFE) mold with slots for multiple samples’ preparation was fabricated to obtain drop-casted films of 50 × 10 × 2 mm (L × W × T) dimensions. A total of 3 mL of each ink was placed into each slot of the mold and then left overnight under a fume hood and under vacuum to evaporate the solvent and obtain rectangular films. Samples were cut in 10 × 10 mm square specimens and imaged under optical microscope (HRX-01, Hirox, Tokyo, Japan) for analyzing the surface profile [30,31,32]. A total of ten 800 µm × 800 µm samples were selected for each formulation to evaluate both roughness and waviness. The roughness was measured through the parameter *S_q_*, defined as the root mean square value of the ordinates within the definition area, and the waviness, defined as the average of the highest difference between peaks and valleys in the profiles of the samples [33].

### 2.5. Statistical Analysis

Statistical analysis was carried out to discuss the significance of the differences observed between the BC/HA and FC/HA blends. Independent *t*-tests were performed with the Jamovi Software (V. 2.2.5) by taking into account the numerosity of the samples and setting a significance threshold equal to 0.05.

## 3. Results

We carried out a complete rheological characterization of the BC/HA and FC/HA slurry-like compounds aimed at evaluating their main rheological properties, namely the amplitude of the complex viscosity (*η*), the storage (*G′*) and loss (*G″*) moduli, and the shear stress (*τ*), as a function of either the strain (*γ*) or the oscillating frequencies (*f*)—therefore the shear rate ($\dot{\gamma }$)—in a time window of 10 days to highlight potential time-dependent trends due to aging. The rheological behavior of the samples showed marked relative differences across time when sweeping either frequency or strain amplitudes. Concerning the frequency sweeps, both FC/HA and BC/HA samples presented a progressive decreasing trend over time of all the measured rheological quantities. The most relevant drop was observed after six days with a further, but limited, reduction in the order of 10% on day 10 from day 6 (see details in Figures S1 and S2 in the Supplementary Materials).

**Figure 1:**
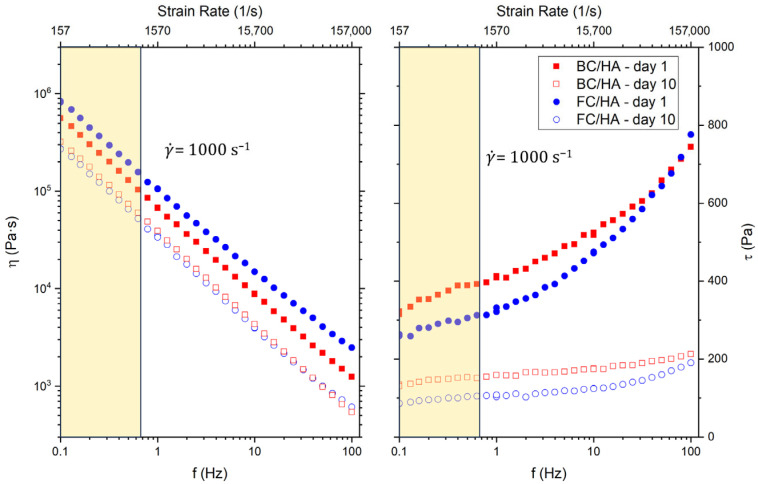
Rheology results as a function of the frequency (*f*) and shear rate ($\dot{\gamma }$) for the BC/HA and FC/HA samples between day 1 and day 10. Complex viscosity (*η*) and shear stress (*τ*) as a function of the frequency (*f*). The yellow shaded area highlights the region of shear rates up to $\dot{\gamma }$ = 1000 s^−1^, typical of 3D printing applications [34].

The results from both the BC/HA and FC/HA samples in the 1–10-day time window for the complex viscosity and the shear stress are reported in Figure 1. For both compositions, on both day 1 and day 10, we observed a drop of three orders of magnitude. The curves on day 10 showed data that almost overlapped. Looking at the viscosity results as a function of the shear rate, considering the region up to 1000 s^−1^, characteristic of nozzle-based 3D printing applications [34], the viscosity results in the order 10^5^ Pa∙s. The shear stress *τ* showed increasing trends, for both the blends, with values always below 1 kPa across the observed frequency range.

**Figure 2:**
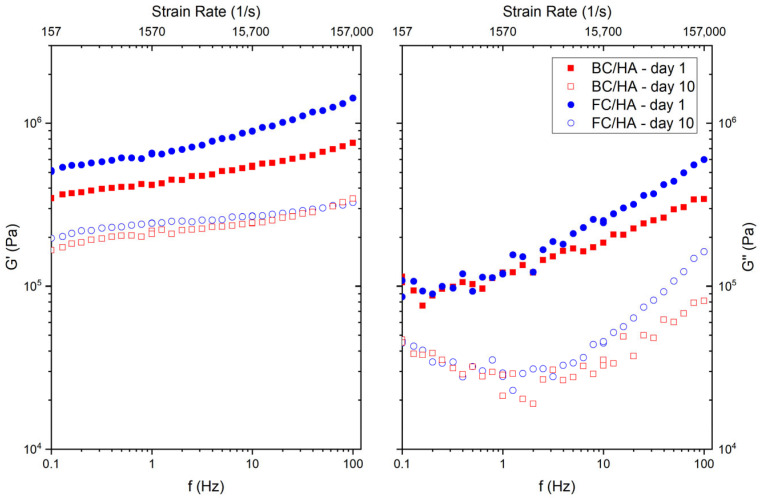
Rheology results as a function of the frequency (*f*) and shear rate ($\dot{\gamma }$) for the BC/HA and FC/HA samples between day 1 and day 10. Storage (*G′*) and loss modulus (*G″*) as a function of the frequency (*f*).

Referring to Figure 2, the storage modulus *G′* presents slight differences between the BC/HA and the FC/HA samples at low frequencies, being 3.5 × 10^5^ Pa (BC/HA, day 1) and 2 × 10^5^ Pa (BC/HA, day 10) versus 5.1 × 10^5^ Pa (FC/HA, day 1) and 1.7 × 10^5^ Pa (FC/HA, day 10) at 0.1 Hz, respectively. However, the differences progressively reduce as the frequency is increased. The loss modulus *G″* of the two slurries, reported in Figure 2, showed similar trends between samples at same time points. On day 1, both slurries have *G″* in the order of 10^5^ Pa at 0.1 Hz with a monotonic growth with the frequency that reaches values of 3.4 × 10^5^ Pa for the BC/HA sample and 6 × 10^5^ Pa for the FC/HA sample at 100 Hz. On day 10, both samples start at 0.1 Hz with values in the order of 4.5 × 10^4^ Pa, reaching 100 Hz values in the order 1.6 × 10^5^ Pa for the FC/HA sample, and 0.81 × 10^5^ Pa for the BC/HA sample.

**Figure 3:**
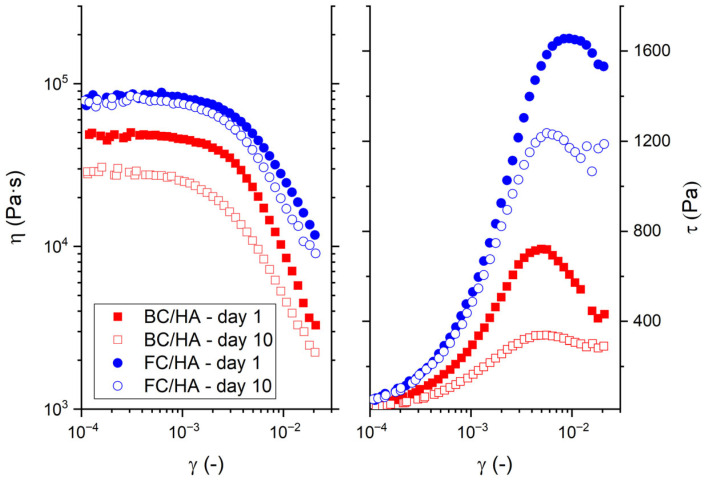
Rheology results as a function of the strain (*γ*) for the BC/HA and FC/HA samples between day 1 and day 10. Complex viscosity (*η*) and shear stress (*τ*) as a function of the strain amplitude (*γ*).

Figure 3 reports the synoptic plots for the rheological properties of the BC/HA and FC/HA samples as a function of the strain amplitude on day 1 and day 10 (details in Figures S3 and S4 in the Supplementary Materials). Regarding the strain sweep characterization, more homogenous trends between the two types of samples are observed. In fact, the complex viscosity *η* for both collagens presents a first plateau at lower strains up to a minimum value of about 0.001, with values gently decreasing at higher strains. However, the respective curves of *η* for the two types of slurries remain well-distinct in their values, with FC/HA resulting in being more viscous than BC/HA over the whole range of strain amplitudes and with reciprocal differences that slightly increase with the strain amplitude. As for the FC/HA slurries, we observe an insensitivity to time at least up to day 10, with values of *η* of about 8 × 10^4^ Pa∙s at *γ* = 10^−4^. In contrast, the BC/HA slurries presented a more pronounced variation in η between days 1 and 10 at every strain, with values of 4.7 × 10^4^ Pa∙s and 2.7 × 10^4^ Pa∙s at *γ* = 10^−4^ respectively for day 1 and day 10. In Figure 3, the shear stress *τ* increased with the strain up to a maximum value, which we identified as the yield stress. While the FC/HA samples presented yield stresses of about 1600 Pa on day 1 and about 1200 Pa on day 10, the BC/HA samples showed differences at the maximum of the curve across time (≈700 Pa vs. ≈380 Pa on days 1 and 10, respectively). The strains associated with the yield stresses, defined as yield strains, were located at similar values for slurries based on different collagens, being about ≈5.5 × 10^−3^ and ≈5 × 10^−3^ for the FC/HA and BC/HA samples, respectively.

**Figure 4:**
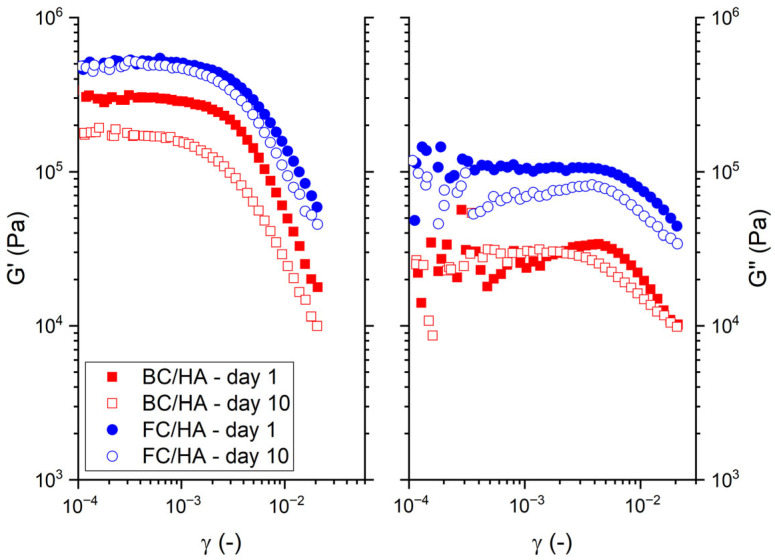
Rheology results as a function of the strain (*γ*) for the BC/HA and FC/HA samples between day 1 and day 10. Storage (*G′*) and loss modulus (*G″*) as a function of the strain (*γ*).

Concerning the storage and loss moduli reported in Figure 4, *G′* had values almost insensitive to time for the FC/HA sample. Looking at the lowest strains, the values of the storage modulus *G′*_0_ were about 5 × 10^5^ Pa for the FC/HA samples and 3 × 10^5^ Pa for the BC/HA samples on day 1. At the same amplitude, but on day 10, the BC/HA sample showed a decrease in *G′*_0_ down to 1.8 ×10^5^ Pa. In contrast, *G″* was almost insensitive to time, at highs strains, only for the BC/HA samples, while the FC/HA samples displayed a general decrease of some tens of kPa through time.

**Figure 5:**
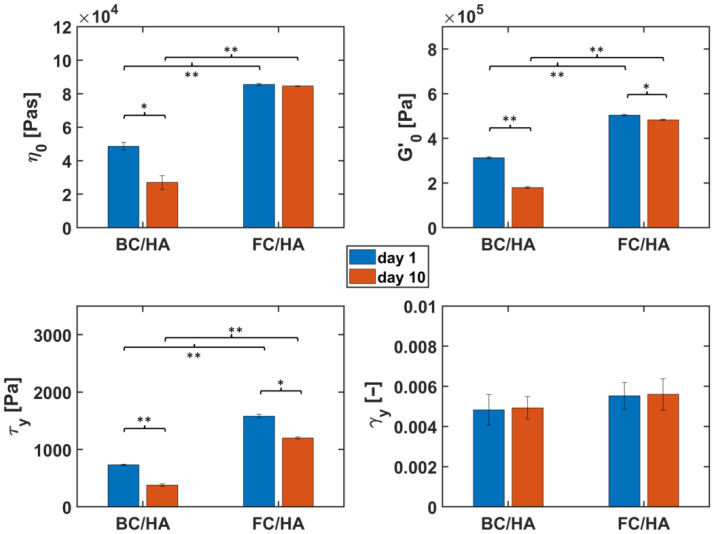
Summary of the main results from the rheology tests and statistical comparison between the datasets from the BC/HA and FC/HA samples on days 1 and 10. (**Top left**): Complex viscosity (*η*); (**Top Right**): Storage (*G′*_0_); (**Bottom left**): Yield stress (*τ_y_*); (**Bottom right**): Yield strain (*γ_y_*). Statistical significance of the comparisons between groups: * *p* < 0.05; ** *p* < 0.001.

To better describe the differences between the slurries and the material sensitivity to time in view of a fabrication involving additive manufacturing, we report in Figure 5 a statistical analysis of the main rheological properties estimated with the experimental tests.

Concerning the viscosity, there was a statistically significant difference only for the BC/HA slurries between the values on days 1 and 10 (*p* < 0.05). In fact, regarding the FC/HA samples, the associated *p*-value is above 0.05. In contrast, statistically significant differences (*p* < 0.001) resulted between the values of BC/HA and FC/HA at both times. The comparisons between the *G′*_0_ values showed statistically significant differences between the observation days for both types of samples (*p* < 0.001 for BC/HA and *p* < 0.05 for FC/HA), in which a decrease in such property was evident. The same outcome was obtained from the comparison between the different samples considered on the same day (either 1 or 10, respectively), with a *p*-value lower than 0.001 in either case. Interestingly, while the yield stress *τ_y_* showed a similar scenario as the *G′*_0_, the analysis of the yield strains led to the conclusion that all the observed differences in *γ_y_*, independent of the selected nature of the sample or observation time, were not statistically significant (*p* > 0.05 in all cases).

**Figure 6:**
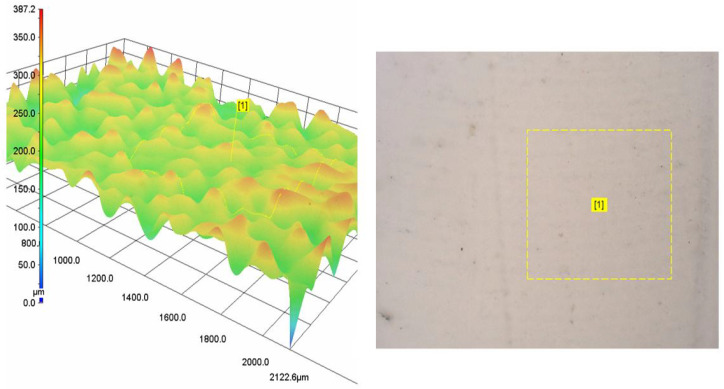
Example of the morphological analysis of drop casted films obtained by BC/HA and FC/HA slurries. In this specific case, the (**left panel**) shows the rendering of the BC/HA surface obtained from the analysis of the specific region, labeled as [1], on the sample highlighted in the (**right panel**) by the dashed line square, obtained through optical microscopy.

Drop casting of the inks resulted in homogeneous adhesion of polymer chains to the mold, and subsequent solvent evaporation caused slight volume contraction due to polymer chain packaging. Both BC/HA and FC/HA films appeared highly opalescent, due to the inclusion of HA within the polymeric matrix, and fragile. Optical microscopy imaging revealed a morphological homogeneity in both cases. Moreover, surface profilometry analysis showed a difference between the two compositions, with a similar parameter *S_q_* equal to 30.3 µm and 27.8 µm for the BC/HA and FC/HA, respectively, but a different waviness equal to 101.3 µm vs. 39.6 µm for the BC/HA and FC/HA, respectively. An example of the outcomes from optical microscopy is shown in Figure 6.

## 4. Discussion

In this work, we aim to describe the rheological behavior of slurries made of two types of animal-based collagen peptides and HA in view of their employment as potential candidates for additive manufacturing applications for bone tissue replacements. The increasing demand of collagen as a constitutive material in biomedical applications has pushed researchers to seek different affordable, safe, and sustainable collagen sources able to fulfill the usual stringent requirements of biocompatibility and mechanical stability [5]. Animal collagen has represented a valuable alternative to human collagen, with remarkable properties which, however, are dependent on the specific source. Nevertheless, although BCs present a high biocompatibility, they are prone to contamination and, due to religious beliefs, are not always accepted by patients [17]. In contrast, FCs have been considered a valuable alternative, although they generally possess inferior mechanical properties than those from mammalians [15].

In recent years, the advent of additive manufacturing technologies, like 3D printing, have revolutionized design principles, giving the possibility to create new material blends and develop complex structures [35]. Collagen derivatives, both from humans or animals, have been used in a number of additive manufacturing applications, especially in the form of blends with HA for creating bone-like tissues [14]. Despite its massive employment, however, a detailed evaluation of the rheological properties of animal-based collagen/HA slurries is still missing. A deeper understanding of such features may help, indeed, to optimize the fabrication processes, leading to the development of advanced constructs able to better fulfill the requirements of final application but also to the investigation of the healing mechanism in collagen-based materials.

Since it has been already demonstrated that there are no major difference in chemical structure between bovine and fish collagen peptides [28], our study delivers a description of the behavior of acidic slurries containing BC/HA and FC/HA and the relative differences as a function of either frequency, in the bandwidth 0.1–100 Hz (corresponding to shear rates up to about 157,000 s^−1^ ), or strain amplitudes, in a range of 0.002–2%, at different aging times between 1 and 10 days. A general observation for all tests concerns the behavior of the nanocomposite slurries over time, leading to the need for a specific design strategy to fabricate structures and predict their mechanical behavior.

The first relevant result is related to the values of the magnitude of complex viscosity that are higher than those from a recent published study (see [36]) for both formulations. In particular, the trend across frequencies is similar but the measured values are about three-orders-of-magnitudes higher (~10^5^–10^6^ vs. ~10^3^ Pa∙s at 0.1 Hz) [36]. This difference could be explained by the differences in the structure of the slurries, which in our case consisted of collagen peptides loaded with HA nanoparticles while in the recalled study were animal-based methacrylated collagens [36]. The difference in the original collagen structure (peptide vs. full chain) and strengthening mechanisms may lead to different mechanical properties in which, as expected, HA influences the final outcomes the most. The magnitude of *G′* and *τ_y_* obtained in our study is therefore closer to that of dense collagen/HA slurries and distinct from softer alginate–HA hydrogels, which typically display yield stresses below 500 Pa and *G′* values in the 10^3^–10^4^ Pa range [14]. The higher viscoelastic moduli of our materials can be attributed to extensive electrostatic and hydrogen-bonding interactions between protonated collagen peptides and HA nanoparticles in the acetic acid environment, forming a percolated microstructure that sustains mechanical load. These quantitative differences emphasize that while HA incorporation enhances stiffness and printability across all formulations, the governing mechanism shifts from matrix-driven (in alginate systems) to interaction-driven (in acetic acid slurries). Overall, our results confirm that the fish-collagen-based nanocomposite displays the best rheological stability (higher *G′*) and structural integrity over time, reinforcing the view that formulation chemistry—particularly solvent polarity and particle dispersion—determines long-term mechanical performance more than the intrinsic collagen source [28].

Moreover, almost flat behavior of G″ within 0.1–10 Hz was observed, with monotonic positive tendency with increasing frequencies, demonstrating how the analyzed materials were able to uniformly dissipate external pulsating loads in a specific low bandwidth. In a different study, Elango et al. investigated fish collagen/HA scaffolds prepared from blue shark cartilage and reported *G′* values between 2 × 10^5^ Pa and 4 × 10^5^ Pa, depending on HA content, demonstrating effective particle–polymer reinforcement and biocompatibility with osteoblast-like cells [22]. Similarly, Lin et al. formulated collagen/HA inks for low-temperature 3D printing that exhibited shear-thinning behavior and G′ values near 1 × 10^5^ Pa across 0.1–10 Hz, ensuring structural fidelity during layer deposition. Their study confirmed that increasing HA concentration above 40 wt% markedly improved the yield stress and print accuracy of collagen-based scaffolds by promoting nanoparticle bridging between fibrils [37].

The outcomes of the rheological assessment are generally one- to two-orders-of-magnitude higher than those observed in alginate-based systems, where the polysaccharide matrix governs viscoelastic behavior and its ionic crosslinking primarily dictates network stiffness. Leukers et al. showed that HA scaffolds printed with biopolymer matrices reached compressive strengths between 1.2 MPa and 2.0 MPa, depending on HA fraction, confirming that particle–matrix integration critically determines structural integrity [38]. More recently, Wierzbicka et al. observed that hybrid alginate–marine-collagen inks exhibited viscosities in the 10^3^–10^4^ Pa·s range upon collagen addition. However, in their system the employment of different collagen sources presented minimal variations in mechanical behavior, since the alginate matrix dominated the viscoelastic response [39]. By contrast, in our acetic acid-based formulations, the absence of ionic crosslinking allowed the intrinsic peptide–particle interactions to emerge more clearly: fish-derived peptides produced a denser, more cohesive network with different rheological decay between collagen sources over time.

In a recent related study, nanocomposites derived from marine and bovine collagens were formulated using sodium alginate as an additional component to HA. Both formulations showed solid-like characteristics (*G′* > *G″*) across the 0.4–100 Hz frequency range, confirming their suitability for extrusion-based bioprinting. The viscosity of the fish- and bovine-based nanocomposites ranged between 10^3^ and 10^5^ Pa·s, displaying the typical shear-thinning behavior of hydrogels. The fish-derived bioink demonstrated slightly higher viscosity and shear resistance, indicating marginally enhanced elastic recovery. However, the differences between the two inks were markedly smaller than those observed in the acetic acid-based systems presented here, suggesting that the viscoelastic response was predominantly dictated by alginate crosslinking and hydroxyapatite dispersion rather than by the peptide source.

Concerning the results from the strain sweep test, the FC/HA samples showed a statistically significantly higher viscosity *η* and stiffness *G′*_0_ than that of the BC/HA blends. These two properties are important in 3D printing applications because they represent the most conditioned ones for an ink during the extrusion process in terms of manufacturability and stability [40]. It is also noteworthy that while the values of the yield stresses *τ_y_* were slightly higher for the FC/HA samples with respect to the BC/HA ones (~1500 Pa vs. ~600 Pa), the yield strains *γ_y_* were statistically similar between the two groups, leading to a different toughness between the blends and thus between the two animal sources.

These results can be compared to other nanocomposites presented in the literature. Milazzo et al. developed HA-based slurries with the addition of silk, using water as solvent: comparing their results, and, in particular, the *G′*_0_, *τ_y_* and *γ_y_*, we noticed that the experiments with collagen/HA slurries, independently of the source, led to values similar to those of HA/water, meaning that collagen, contrary to silk, did not significantly affect the mechanical properties of the blend, thus leaving HA as the main contribution to the stability and toughness of the material. From a fabrication standpoint, it was desired to have similar results as the HA/water slurries achieved in other works [38,40,41], characterized by high quality and stability. However, the presence of collagen, like in our slurries, is important from a biological perspective since it is known to enhance those cellular functions that lead to a better integration of the constructs in prosthetic applications [37,42,43,44].

The employment of drop casting presented in this work aims at delivering a simple, but effective, application of the nanocomposite slurries in the framework of additive manufacturing. From the morphological analysis of the surfaces of the samples, these two collagen-based biomaterial samples share a similar surface topography (i.e., comparable micro-roughness) but differ in waviness; the BC/HA with higher waviness may better promote contact guidance, spatial cell alignment, and more anisotropic extracellular matrix deposition because subtle undulations can serve as physical cues guiding cytoskeletal organization [45,46]. At the same time, increased waviness can lead to nonuniform stress distribution under mechanical load, possibly causing local strain concentrations or delamination of layers in composite scaffolds. The FC/HA smoother-wavy sample, on the other hand, would tend to provide more uniform mechanical behavior and more homogeneous cell coverage, but may lack sufficient directional cues to influence cell orientation or patterned matrix formation. For example, collagen scaffolds with oriented micro-architectures have been shown to drive aligned ECM secretion and improved tissue regeneration compared to isotropic ones [47,48].

In future work, we plan to use the information gathered in this study to properly select the best 3D printing approach to fabricate samples. According to previous studies, potential applications for collagen and collagen/HA would require either extrusion-based or jetting-based printing methodologies in which the slurry can be ejected, in the form of a filament or droplet, through a mechanical push given by either pressurized air or a mechanical actuation of plunging [49,50,51]. A different approach, in contrast, would consist of using stereolithography, but the ink has to be provided a photo-crosslinkable agent able to react to ultraviolet light (UV) [52]. All in all, the final selection of the 3D printing technique will be defined by the targeted application and specifically by the level of accuracy and precision required to meet the functional requirements.

## 5. Conclusions

This study systematically compared the rheological behavior and morphological features of bovine- and fish-derived collagen peptide slurries incorporating hydroxyapatite in acetic acid, with the goal of assessing their suitability as potential bioinks for bone tissue engineering. Both formulations exhibited viscoelastic properties and yield stress values comparable to those reported for aqueous hydroxyapatite-based inks already proven suitable for 3D printing. The fish collagen-based slurries demonstrated higher viscosity and greater stiffness compared to their bovine counterparts. Despite these differences, both materials maintained shear-thinning behavior within the range of shear rates typical of extrusion-based additive manufacturing processes.

The morphological analyses confirmed that both blends produced homogeneous surfaces, though bovine-based films displayed higher waviness, which could influence cell alignment and mechanical response in future biological evaluations. Overall, the rheological profiles and morphological properties suggest that both formulations are promising candidates for extrusion-based 3D printing of collagen–hydroxyapatite composite scaffolds, with fish collagen showing superior stability and mechanical consistency. Future work will focus on overcoming the current limitations of this study, including the optimization of printing parameters and crosslinking strategies, as well as on the biological assessment of the printed constructs to confirm their applicability in bone tissue regeneration.

## Data Availability

Data are available upon request to the corresponding authors. The original contributions presented in this study are included in the article/Supplementary Material. Further inquiries can be directed to the corresponding authors.

## Supplementary Materials

The following supporting information can be downloaded at: https://www.mdpi.com/article/10.3390/polym17233196/s1, Figure S1: Rheological properties as a function of frequency for the FC/HA samples between day 1 and day 10. Figure S2: Rheological properties as a function of frequency (*f*) for the BC/HA samples between day 1 and day 10. Figure S3: Rheological properties as a function of strain (*γ*) for the FC/HA samples between day 1 and day 10. Figure S4: Rheological properties as a function of strain (*γ*) for the BC/HA samples between day 1 and day 10. All Figures S1–S4 report the results for complex viscosity (*η*), shear stress (*τ*), and storage (*G′*) and loss (*G″*) moduli.

## Abbreviations

The following abbreviations are used in this manuscript:

BC	Bovine Collagen
FC	Fish Collagen
HA	Hydroxyapatite
