# Supplementary material for: Rheological Characterization of Marine and Bovine Collagen Peptides/Acetic Acid Slurries Incorporating Hydroxyapatite Nanoparticles
Source: Polymers (Basel). 2025 Nov 30;17(23):3196. doi: 10.3390/polym17233196 (PMC12693822; doi:10.3390/polym17233196)
Supplement: Supplementary file 1 [file polymers-17-03196-s001.zip › polymers-3985790-supplementary.pdf]

Supplementary Materials

Rheological Characterization of Marine and Bovine Collagen Peptides/Acetic Acid Slurries Incorporating Hydroxyapatite Nanoparticles

Mario Milazzo [1,2,*], Claudio Ricci [1], Eugenio Redolfi Riva[2], Damiano Rossi[1], Irene Anguillesi[1], Maurizia Seggiani [1], Giuseppe Gallone [1], and Serena Danti [1,*]

[1] Department of Civil and Industrial Engineering, University of Pisa, Largo Lucio Lazzarino, 56126 Pisa, Italy; claudio.ricci@unipi.it (C.R.); irene.anguillesi@unipi.it (I.A.); giuseppe.gallone@unipi.it (G.G.); maurizia.seggiani@unipi.it (M.S.);
[2] The BioRobotics Institute, Sant'Anna School of Advanced Studies, Viale Rinaldo Piaggio, 56025 Pontedera, Italy; eugenio.redolfiriva@santannapisa.it (E.R.R.)
* Correspondence: mario.milazzo@unipi.it (M.M.), serena.danti@unipi.it (S.D.)

In the following are reported the experimental results from rheological characterizations carried out on samples of either Marine (FC) and Bovine (BC) Collagen Peptides/Acetic Acid Slurries incorporating Hydroxyapatite Nanoparticles (HA).

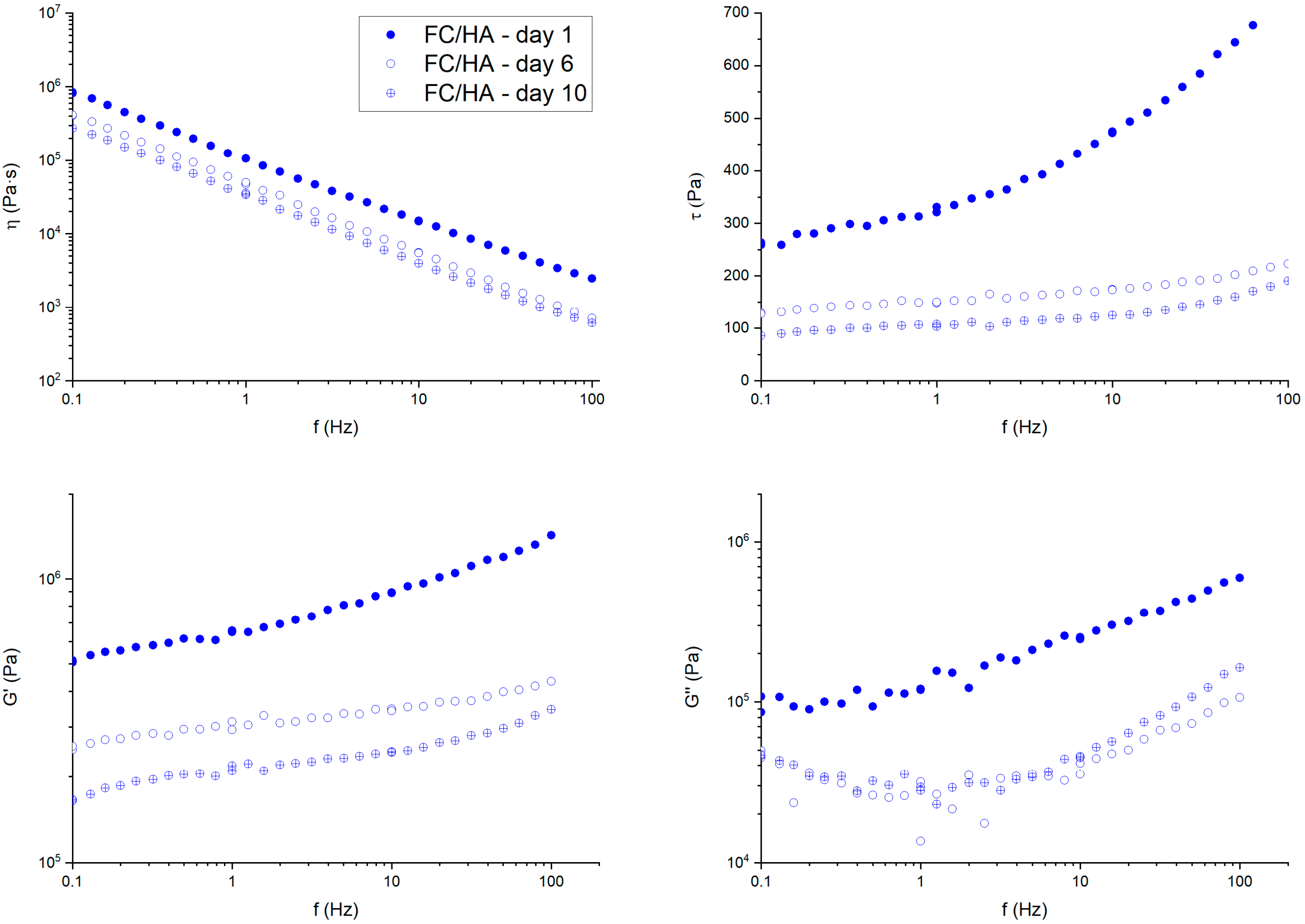

Figure S1 Rheological properties as a function of frequency (*f*) for the FC/HA samples between day 1 and day 10. From left to right and from top to bottom: complex viscosity (η), shear stress (τ), storage (G') and loss (G'') moduli.

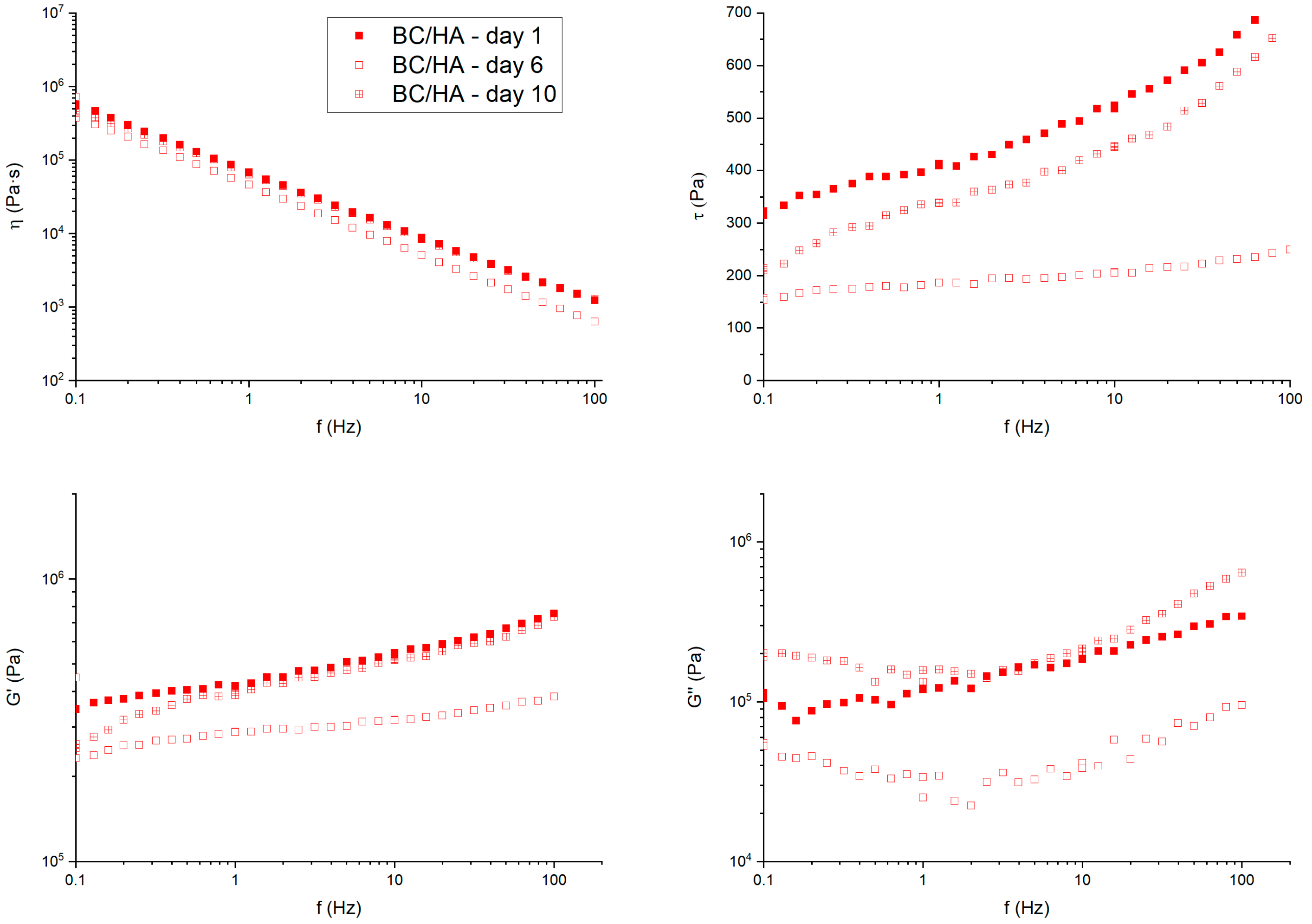

Figure S2. Rheological properties as a function of frequency (*f*) for the BC/HA samples between day 1 and day 10. From left to right and from top to bottom: complex viscosity (η), shear stress (τ), storage (G') and loss (G'') moduli.

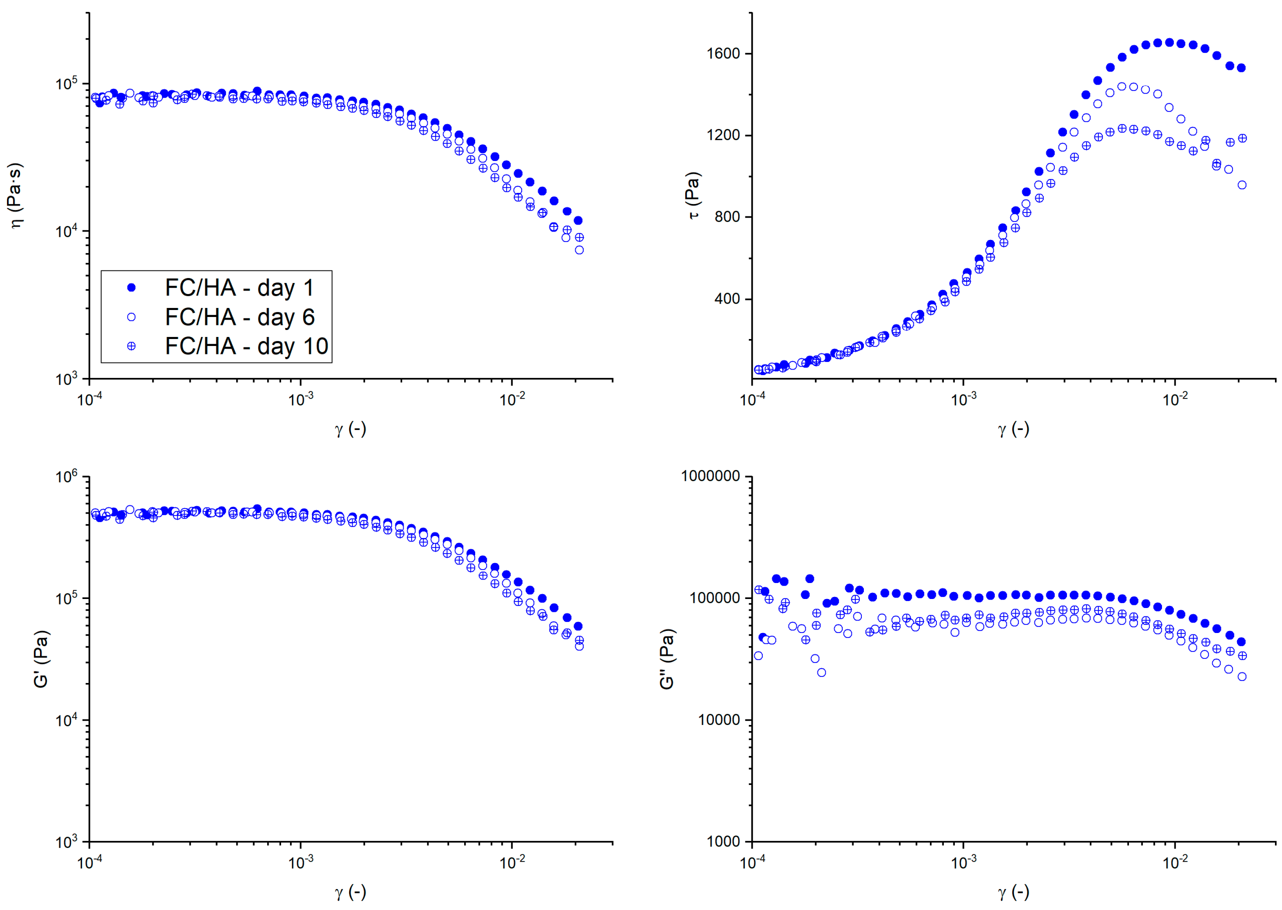

Figure S3. Rheological properties as a function of strain (γ) for the FC/HA samples between day 1 and day 10. From left to right and from top to bottom: complex viscosity (η), shear stress (τ), storage (G') and loss (G'') moduli.

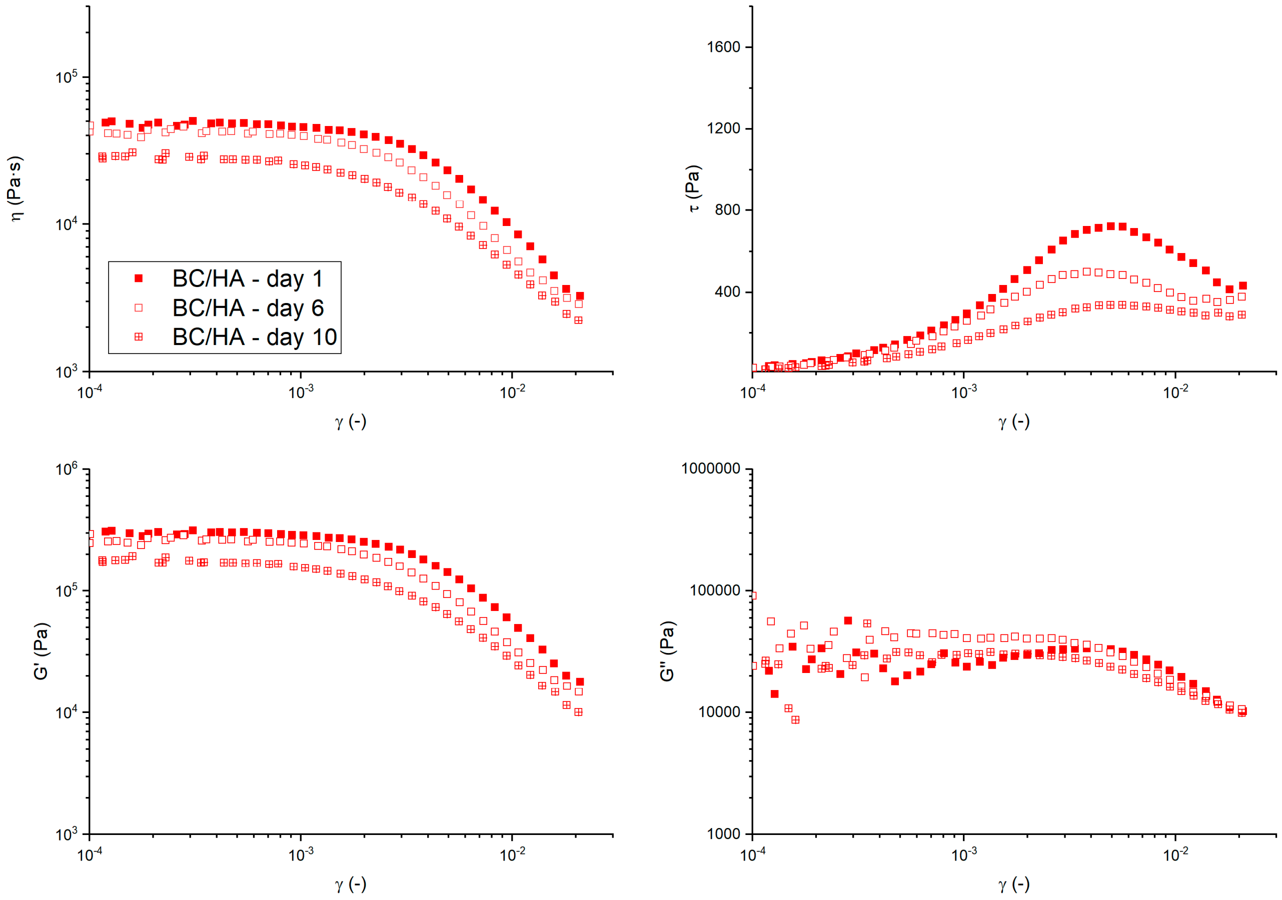

Figure S4. Rheological properties as a function of strain (γ) for the BC/HA samples between day 1 and day 10. From left to right and from top to bottom: complex viscosity (η), shear stress (τ), storage (G') and loss (G'') moduli.
